# Case Report: Clinical presentations of cyanosis associated with acquired methemoglobinemia in infants—a clinical challenge

**DOI:** 10.3389/fped.2025.1563277

**Published:** 2025-05-13

**Authors:** Lin Wang, Yang Ye, Delian Li, Kexing Li, Peng Yang, Huanrui Hu, Xiaoliang Liu, Yongmei Zhao

**Affiliations:** ^1^ Department of Pediatrics, Longquanyi District of Chengdu Maternity & Child Health Care Hospital, Chengdu, Sichuan, China; ^2^ Division of Vascular Surgery, Department of General Surgery, West China Hospital, Sichuan University, Chengdu, China; ^3^ Key Laboratory of Birth Defects and Related Diseases of Women and Children (Sichuan University), Ministry of Education, Department of Pediatrics, West China Second University Hospital, Chengdu, China; ^4^ Department of Pediatrics, West China Second University Hospital, West China Second University Hospital-Tianfu·Sichuan Provincial Children’s Hospital, Sichuan, China

**Keywords:** cyanosis, acquired methemoglobinemia, infant, methylene blue, nitrate

## Abstract

**Background:**

Cyanosis is a common clinical finding in infants and children. Particularly, central cyanosis can be associated with significant and potentially life-threatening diseases. Acquired methemoglobinemia is a rare but severe or even fatal cause of cyanosis in infants. Due to its rarity, timely diagnosis and appropriate management, particularly in infants, can be challenging in a clinical setting.

**Case report:**

We report the case of a previously healthy 49-day-old female infant who presented with central cyanosis. Four hours prior to presentation, her guardian had inappropriately prepared her milk formula using spinach juice. This critical clue hinted to us that this infant might suffer from acquired methemoglobinemia. Upon blood sampling, her blood appeared chocolate brown in color. Furthermore, arterial blood gas analysis revealed abnormal findings, with a significantly elevated percentage of methemoglobinemia at 44.7%. Regarding the history of inappropriate formula preparation using vegetable juice and the abnormal finding of methemoglobinemia, a diagnosis of acquired methemoglobinemia was proposed. Other causes of methemoglobinemia were further excluded. Treatment with methylene blue and vitamin C was immediately initiated. Encouragingly, the cyanosis of this infant resolved 1 h later, with normal results of the repeated blood gas analysis. This infant was discharged home 2 days later and had no abnormal findings during the follow-up.

**Conclusion:**

In this study, we reported a rare case of acquired methemoglobinemia in a 49-day-old infant. Inappropriate preparation of the infant milk formula with spinach juice was the potential cause of methemoglobinemia in this case, which presented with central cyanosis. Our findings also suggested that pediatricians should be aware of acquired methemoglobinemia as a potential cause of cyanosis in infants.

## Introduction

Cyanosis is a bluish-purple discoloration of the tissues caused by an increased concentration of deoxygenated hemoglobin in the capillary bed, resulting from various conditions (1). This condition is a common clinical finding in infants and children. Particularly, central cyanosis can be associated with significant and potentially life-threatening diseases. Therefore, it is crucial to identify the underlying causes of cyanosis in the clinical setting, which is essential for appropriate management. The causes of cyanosis in infants include respiratory, pulmonary, cardiac, metabolic, neurologic, infectious, and hematologic disorders (2). Respiratory causes most often account for previously healthy infants with the new onset of central cyanosis (1). Cardiac and pulmonary vascular etiologies might also be responsible for cyanosis, particularly in young infants. Moreover, other severe conditions produce central cyanosis through disordered breathing caused by neurologic disease or decreased ability for hemoglobin to carry oxygen, such as methemoglobinemia.

Methemoglobinemia is a rare disorder associated with the oxidation of Fe^2+^ of hemoglobin (Hb) to Fe^3+^ of methemoglobin. The presence of the Fe^3+^ state results in allosteric changes that irreversibly permit oxygen binding. As a result, the corresponding ferroglobins in the tetramer shift the oxygen dissociation curve of Hb to the left. This shift leads to increased affinity of the ferrous iron for oxygen, thus impairing oxygen release to the tissue, resulting in hypoxia with the so-called functional anemia without a decrease in Hb. Regarding the causes of methemoglobinemia, it can be classified into congenital or acquired forms. Congenital methemoglobinemia is caused by autosomal recessive variants in the CYB5R3 gene and autosomal dominant variants in the globin genes known as HbM disease (3–5). Patients with congenital methemoglobinemia are asymptomatic except for cyanosis, but some patients may have severe morbidity which can be fatal in neonates. As the most common form of methemoglobinemia, patients with acquired methemoglobinemia mainly suffer from various exogenous substances that increase methemoglobin formation, including dapsone, antimalarial agents, topical anesthetics, inhaled nitric oxide, rasburicase, nitrates, and nitrites (5–14). Notably, acquired methemoglobinemia can be severe or even fatal, depending on the proportion of methemoglobin. Given the rarity of this disorder, the diagnosis of methemoglobinemia often remains delayed in clinical settings; therefore, the appropriate management, particularly in infants, may be challenging (5, 15).

Herein, we report a rare case of acquired methemoglobinemia in a 49-day-old infant who presented with central cyanosis, attributed to inappropriate preparation of milk formula with spinach juice. Pediatricians need to recognize acquired methemoglobinemia as a potential cause of cyanosis in infants.

## Case presentation

**Figure 1:**
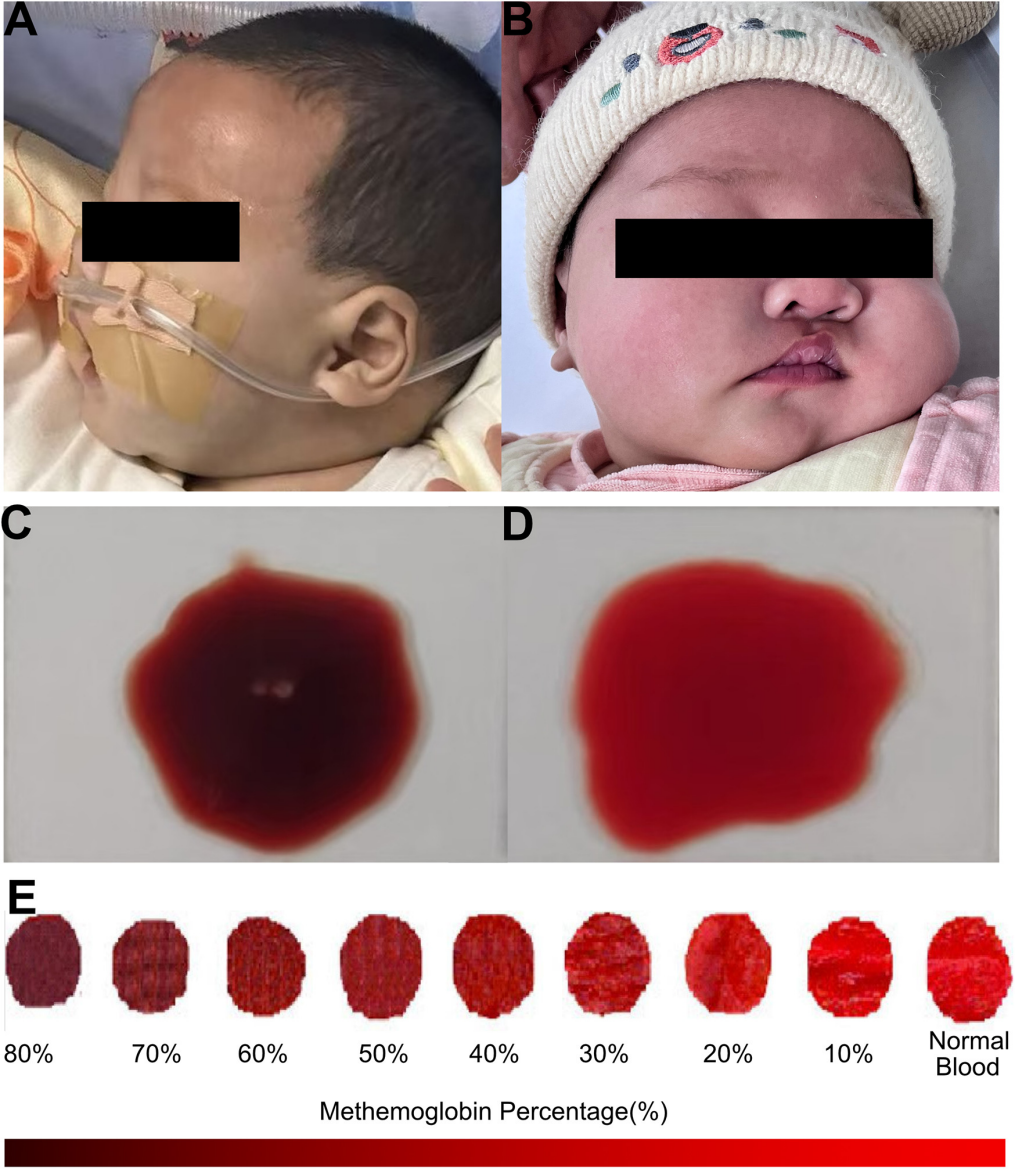
Clinical findings of the infant with acquired methemoglobinemia. **(A)** This infant presented with central cyanosis **(B)** and had no abnormal findings during the follow-up. **(C)** Blood presented with a chocolate-brown color. **(D)** Blood color was normal when clinical conditions recovered. **(E)** An obvious change of blood in color from red to dark brown among patients with methemoglobinemia, reproduced with the free permission of Copyright © 2009 American College of Emergency Physicians (16).

A previously healthy 49-day-old female (Chinese, Han ethnicity) was admitted to the pediatric emergency department of our hospital with her parents reporting she presented with cyanosis persisting for 4 h. She was immediately transferred to our inpatient department under nasal cannula oxygen inhalation. On arrival, the physical examination revealed a body temperature of 36.0°C, heart rate of 158 beats per minute, blood pressure of 125/62 mmHg, respiratory rate of 54 per minute, and initial SaO_2_ of 77%. Central cyanosis was noted, which was evident in the lips, nail beds, earlobes, and mucous membranes (Figure 1A). Capillary refill time and skin temperature were normal. The skin exhibited normal elasticity, without cutaneous mottling, petechiae, and ecchymoses. Pupils were sensitive to light reflex, the anterior fontanelle was flat, and the sign of three depressions was negative. On auscultation, heart sounds were normal, with no murmurs. Neurological examination revealed no signs of meningeal irritation or positive Babinski reflexes, and both patellar and ankle reflexes were normal. No other abnormalities were found in the physical examinations. Moreover, no apparent abnormalities were found during her fetal period. After her birth, the infant had good formula feeding and normal growth and development. The infant had not been exposed to any suspected drugs or toxins, did not have diarrhea, or was undernourished. Her neonatal screening was normal, including G6PD deficiency. Her mother denied a history of respiratory, cardiovascular, and neurological disease, and no family members had a history of related diseases. At the same time, the people living in the same household have shown no relevant clinical manifestations. Notably, 4 h ago, this infant's guardian inappropriately prepared the infant's milk formula with spinach juice. The spinach juice was prepared in advance and kept in the refrigerator for 12–24 h. The mean level of nitrate concentration in *Spinacia* in our area was 1–2 mg/kg. This critical clue hinted that this infant might suffer from acquired methemoglobinemia, associated with nitrates and nitrites from vegetable juice, which increases methemoglobin formation. Moreover, her blood presented with a chocolate-brown color (Figure 1C) when drawn for laboratory tests, compared with its color when clinical conditions recovered (Figure 1D) and consistent with the color change of blood among patients with methemoglobinemia (Figure 1E) (16).

Furthermore, laboratory tests showed abnormal findings of arterial blood gas analysis, including a significantly elevated percentage of methemoglobinemia at 44.7% (normal range, 0%–6%) and decreased level of Hb was 91 g/L (normal range, 120–145 g/L), with a normal level of pH value, partial pressure of carbon dioxide (PaCO_2_), partial pressure of oxygen (PaO_2_), bicarbonate (HCO_3_^−^), and base excess or deficit. The diagnosis of acquired methemoglobinemia was proposed, based on the history of inappropriately preparing the infant formula using vegetable juice and the abnormal finding of methemoglobinemia. Although clinicians must identify a hereditary cause for methemoglobinemia, the treatment of the acute patient must be initiated as soon as possible which does not require the distinction between acquired and inherited causes at the same time (17). Therefore, the emergency treatment of methylene blue was immediately initiated with a dose of 1.5 mg/kg, in combination with vitamin C (1,000 mg twice a day) and nasal cannula oxygen inhalation (0.5 L/min). Encouragingly, the cyanosis of this infant resolved 1 h later. At the same time, other causes of acquired methemoglobinemia were further excluded by the unremarkable findings of blood routine test, C reaction protein (CRP), erythrocyte sedimentation rate (ESR), procalcitonin (PCT), liver function, renal function, blood creatase levels, blood glucose, blood ammonia, and blood fat. In addition, other causes of cyanosis in infants were further excluded, such as respiratory causes (such as upper airway obstruction, bronchiolitis, pneumonia, pneumothorax), circulatory causes (congenital heart disease, pulmonary hypertension, pulmonary hemorrhage), neurologic causes, by negative findings of chest x-ray, echocardiography, electrocardiogram, and physical examination of the respiratory, cardiovascular, and neurological system. Moreover, efforts were made to classify her methemoglobinemia as the acquired form as her family members did not present with a long-life history of cyanosis, dusky skin, or blue sclera, which might suggest the congenital form. Regrettably, her parents refused whole-exome sequencing.

Encouragingly, the cyanosis of this infant resolved 1 h later after the therapy of methylene blue and Vitamin C was carried out. The repeated blood gas analysis also recovered to normal. In turn, the outcome of this infant also confirmed our primary diagnosis of acquired methemoglobinemia. During the follow-up, this infant was discharged home 2 days later and had no abnormal findings of skin color, arterial blood gas, or blood routine test (Figure 1B).

## Discussion

Methemoglobinemia is a rare cause of cyanosis in children, and clinicians have widely recognized its clinical manifestations. However, this disorder is rarely found in infants, especially those younger than 6 months old. Notably, the clinical presentations of younger infants with acquired methemoglobinemia might be more severe. They had low stomach acid production, large numbers of nitrite-reducing bacteria, the relatively easy oxidation of fetal hemoglobin, and immaturity of the methemoglobin reductase system (18–20). In this study, we described a rare case of a 49-day-old infant who developed acquired methemoglobinemia, presenting with central cyanosis, due to the inappropriate preparation of her milk formula with spinach juice. Encouragingly, her clinical conditions resolved 1 h after receiving the therapy of methylene blue and Vitamin C. Two days later, she was discharged home and had no abnormal findings during the follow-up.

**Figure 2:**
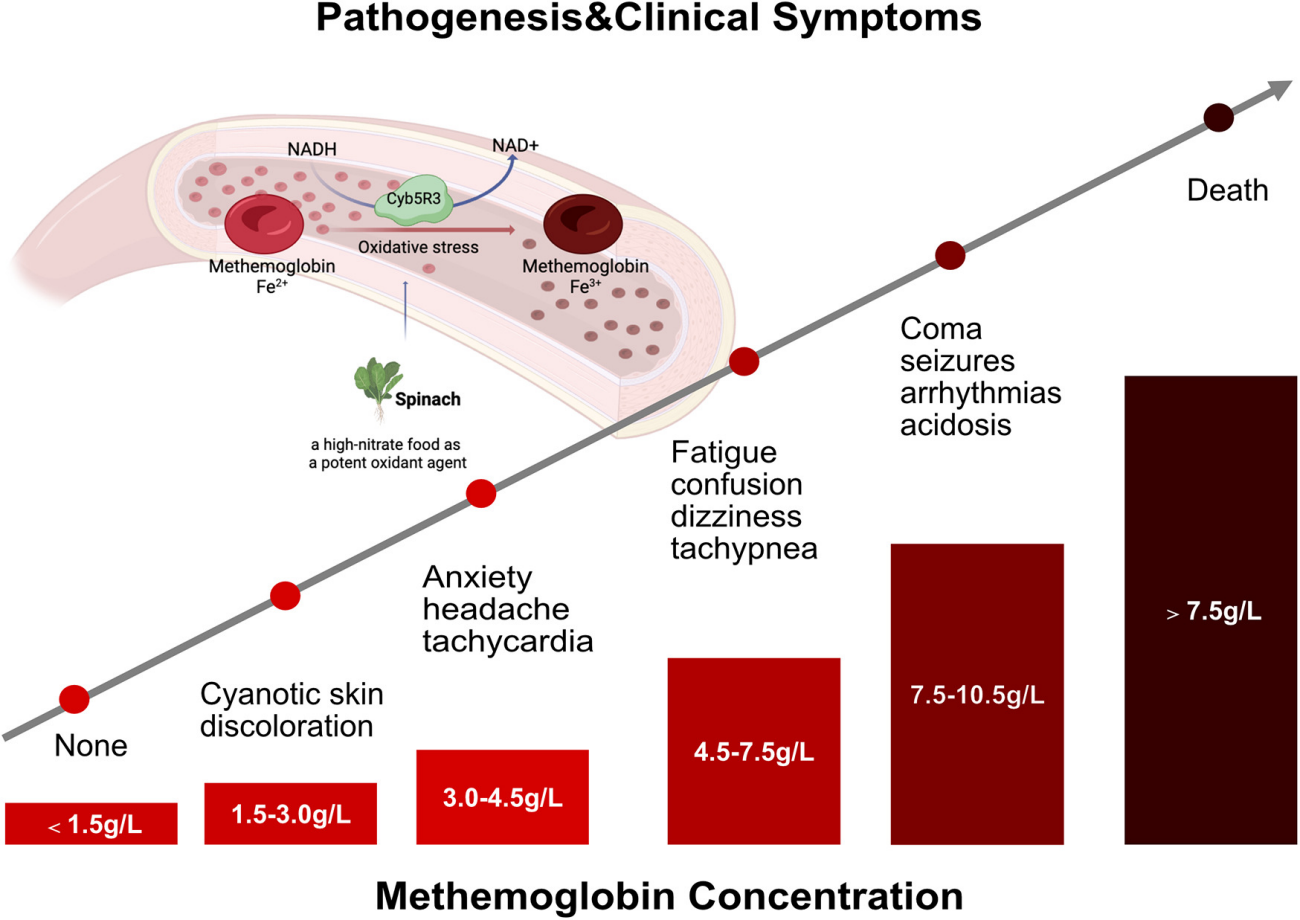
Clinical characteristics of patients with methemoglobinemia. The pathogenesis and clinical manifestations of patients with methemoglobinemia in line with methemoglobinemia concentration.

Regarding the pathogenic basis of methemoglobinemia, the detailed information of clinical and family history, environmental and drug exposure, and evaluation of consanguinity is critical for making the diagnosis, and differential diagnosis between hereditary and acquired forms. The most common form of methemoglobinemia is acquired, associated with various conditions, including diarrhea and acidosis (21, 22) and consumption of high-nitrate water (23, 24) or high-nitrate food (spinach, carrots, beets) (20). Additionally, food-borne nitrates and nitrites exposure to certain drugs can contribute to developing this order. The relevant drugs encompass topical anesthetic agents, silver nitrate, chloroquine, sulfonamides, dapsone, phenacetin, sodium valproate, phenazopyridine, inhaled nitrous oxide, and amyl nitrite (25, 26). Infants with acquired methemoglobinemia are more likely to be exposed to nitrate, including milk formula prepared with healthy water, vegetables containing high nitrate concentration, oxidant drugs, and diarrhea. Upon the ingestion of nitrates, fecal organisms transformed nitrates into nitrites. Subsequently, nitrites are rapidly absorbed from the intestine through passive diffusion and enter the systemic circulation without undergoing the first-pass metabolism in the liver. Notably, nitrites are one of the most potent oxidant agents of ferrohemoglobin (Figure 2). Particularly, nitrate-induced methemoglobinemia is more likely to attack infants younger than 6 months old. It was speculated that these infants produced low levels of stomach acid and had amounts of nitrite-reducing bacteria. In addition, infants have higher levels of fetal hemoglobin, presenting with relatively easy oxidation than adult hemoglobin, and their methemoglobin reductase system remains immature with lower levels of erythrocyte CYB5R activity (18–20). Considering our case, she was previously healthy without infectious disease, diarrhea, or drug administration and did not have G6PD deficiency. Moreover, no nitrite poisoning was found in her family members and people living nearby, which excluded the possibility of consumption of high-nitrate water. It was confirmed that the guardian inappropriately prepared the infant’s milk formula with spinach juice, which is considered a high-nitrate food. Therefore, we speculated that the cause of acquired methemoglobinemia in this case was most likely attributed to the spinach juice-mixed milk formula. The possibility of congenital methemoglobinemia for our case was partly excluded due to the sudden onset of cyanosis and the absence of a long-life history of cyanosis, dusky skin, or blue sclera in the family members. Regrettably, her parents refused whole-exome sequencing. In addition, clinicians needed to identify the causes of cyanosis in neonates and infants, including respiratory, circulatory, neurologic, infectious, and hematologic disorders (2). Respiratory causes most often account for previously healthy infants with the new onset of central cyanosis (1), such as upper airway obstruction, bronchiolitis, pneumonia, and pneumothorax. Circulatory etiologies might also be responsible for cyanosis, particularly in young infants, such as congenital heart disease, pulmonary hypertension, and pulmonary hemorrhage. Moreover, other severe conditions produce central cyanosis through disordered breathing caused by neurologic disease or decreased ability for hemoglobin to carry oxygen. These disorders should be evaluated by detailed clinical symptoms; physical examination of the respiratory, neurologic, and cardiovascular systems; past medical history; a related family history of the disease; and targeted auxiliary examination such as pulmonary imaging, echocardiography, and electrocardiogram.

Recently, a multicenter study reported that the incidence of methemoglobinemia was much lower at 0.015‰; however, the overall 30-day mortality of patients with methemoglobinemia was 6.1% (6). Therefore, clinicians needed to identify the methemoglobinemia and initiate prompt management in children, especially in younger infants. Patients with methemoglobinemia might present with different clinical features in the clinical setting. However, the familiar clinical characteristics of methemoglobinemia are cyanosis, cardiac and neurologic complications, and metabolic acidosis (17, 27). Notably, the clinical symptoms of methemoglobinemia are mainly associated with methemoglobin concentration and methemoglobin level (% of total hemoglobin in patients with nonanemic). Wright et al. (28) concluded that patients with methemoglobin of 1.5 g/dl (10%–20% of total hemoglobin) might develop cyanosis. With the increased concentration and level of methemoglobin, patients gradually developed anxiety, headache, dizziness, fatigue, confusion, tachypnea, arrhythmias, acidosis, seizures, coma, and even death (Figure 2). In this study, the percentage of methemoglobinemia in our infant was significantly elevated to 44.7%, presenting with cyanosis, tachycardia, and high blood pressure. Encouragingly, these clinical symptoms resolved soon after the standard treatment of methylene blue and vitamin C was initiated with this infant.

The management of methemoglobinemia depends on clinical symptoms, methemoglobin and hemoglobin saturation percentage, and underlying conditions. First, the clinician should stop and/or remove the causative agent. In asymptomatic patients who had methemoglobin levels <20%, no treatment was usually needed as the methemoglobin could be reduced by normally metabolizing red blood cells within several hours. Otherwise, interventions should be initiated with the therapy of methylene blue and vitamin C (27). Methylene blue is the cornerstone therapy of methemoglobinemia as it accepts an electron from nicotinamide adenine dinucleotide phosphate (NADPH), reducing Fe^3+^ back to Fe^2+^ in erythrocytes. Its initial dose is 1–2 mg/kg infused intravenously over 3–5 min. It can be repeated at a dose of 1 mg/kg if the clinical conditions do not significantly resolve within 1 h. As a natural water-soluble vitamin, vitamin C reduces excessive oxidative stress, with various doses in children (0.5 g–1.0 g every 12–4 h). In addition, the additional treatment of blood transfusions, exchange transfusion, hemodialysis, and hyperbaric oxygen were also described in patients with refractory methemoglobinemia (17, 27).

## Conclusion

Acquired methemoglobinemia might also involve infants younger than 3 months old. In this case, inappropriately preparing the infant milk formula with spinach juice was the potential cause of methemoglobinemia, which was present with central cyanosis. Pediatricians need to recognize acquired methemoglobinemia as a potential cause of cyanosis in infants.

## Data Availability

The original contributions presented in the study are included in the article/Supplementary Material, further inquiries can be directed to the corresponding authors.
